# Angiotensin-converting enzyme inhibitors and angiotensin-receptor blockers and the risk of COVID-19 infection or severe disease: Systematic review and meta-analysis

**DOI:** 10.1016/j.ijcha.2020.100627

**Published:** 2020-08-27

**Authors:** Daniel Caldeira, Mariana Alves, Ryan Gouveia e Melo, Pedro Silvério António, Nélson Cunha, Afonso Nunes-Ferreira, Luisa Prada, João Costa, Fausto J Pinto

**Affiliations:** a Centro Cardiovascular da Universidade de Lisboa (CCUL), Faculdade de Medicina, Univerisdade de Lisboa, Avenida Professor Egas Moniz, 1649-028 Lisboa, Portugal; b Cardiology Department, Hospital Universitário de Santa Maria (CHULN), Avenida Professor Egas Moniz, 1649-028 Lisboa, Portugal; c Laboratory of Clinical Pharmacology and Therapeutics, Faculty of Medicine, University of Lisbon, Avenida Professor Egas Moniz, 1649-028 Lisboa, Portugal; d Serviço de Medicina III, Hospital Pulido Valente (CHULN), Lisboa, Portugal; e Instituto de Medicina Molecular, Faculty of Medicine, University of Lisbon, Lisboa, Portugal; f Vascular Surgery Department, Hospital Santa Maria, Centro Hospitalar Universitário Lisboa Norte (CHULN), Avenida Professor Egas Moniz, 1649-028 Lisboa, Portugal

**Keywords:** Coronavirus, SARS-CoV-2, Angiotensin-converting enzyme inhibitor, Angiotensin-receptor blocker, Acute respiratory distress syndrome, Acute lung injury

## Abstract

**Objective:**

Animal studies suggested that angiotensin-converting enzyme inhibitors (ACEi) and angiotensin-receptor blockers (ARB) facilitate the inoculation of potentially leading to a higher risk of infection and/or disease severity. We aimed to systematically evaluate the risk of COVID-19 infection and the risk of severe COVID-19 disease associated with previous exposure to (ACEi) and/or ARB).

**Methods:**

MEDLINE, CENTRAL, PsycINFO, Web of Science Core Collection were searched in June 2020 for controlled studies. Eligible studies were included and random-effects meta-analyses were performed. The estimates were expressed as odds ratios (OR) and 95% confidence intervals (95%CI). Heterogeneity was assessed with I^2^ test. The confidence in the pooled evidence was appraised using the GRADE framework.

**Results:**

Twenty-seven studies were included in the review. ACEi/ARB exposure did not increase the risk of having a positive test for COVID-19 infection (OR 0.99, 95%CI 0.89–1.11; I^2^ = 36%; 5 studies, GRADE confidence moderate). The exposure to ACEi/ARB did not increase the risk of all-cause mortality among patients with COVID-19 (OR 0.91, 95%CI 0.74–1.11; I^2^ = 20%; 17 studies; GRADE confidence low) nor severe/critical COVID-19 disease (OR 0.90, 95%CI 0.74–1.11; I^2^ = 55%; 17 studies; GRADE confidence very low). Exploratory analyses in studies enrolling hypertensive patients showed a association of ACEi/ARB with a significant decrease of mortality risk.

**Conclusions:**

ACEi/ARB exposure does not seem to increase the risk of having the SARS-CoV-2 infection or developing severe stages of the disease including mortality. The potential benefits observed in mortality of hypertensive patients reassure safety, but robust studies are required to increase the confidence in the results.

## 1 Introduction

The novel acute respiratory syndrome coronavirus 2 (SARS-CoV-2) firstly identified in Wuhan China lead to a world-wide outbreak pandemic situation with more than 350,000 related deaths [1]. The SARS-CoV-2 goes into the host cells through the angiotensin-converting enzyme (ACE) 2 (ACE2) receptor [2]. Some animal studies showed that angiotensin-converting enzyme inhibitors (ACEi) and angiotensin-receptor blockers (ARB) increase the ACE2, creating the hypothesis that these drugs could facilitate the inoculation of SARS-CoV-2 potentially leading to a higher risk of infection and/or disease severity [3]. The fragility of these assumptions led several medical societies to issue a recommendation for not withdrawing these drugs because the evidence was not compelling and due to the potential harms, as these drugs are effective treatments in the management of hypertension, diabetes mellitus, coronary heart disease, cerebrovascular disease and/or chronic kidney disease for many people. In this systematic review we aimed to assess the risk of infection by SARS-CoV-2 and the risk of mortality or respiratory complications in patients with symptomatic disease of SARS-CoV-2 (COVID-19) related to previous use of ACEi or ARBs.

## 2 Methods

This systematic review followed the reporting principles of MOOSE and PRISMA [4], [5]. The protocol is available at https://osf.io/6vf2w. Patients and public were not involved in this review.

### 2.1 Eligibility criteria

We included all controlled studies with information about risk of infection or the risk of disease complications associated with ACEi and/or ARBs.

For randomized controlled trials or cohort/nested case-control studies that evaluated the risk of infection (positive test), studies had to enrol a population submitted to tests and to report the risk of having a positive test associated with ACEi and/or ARB, or having raw data that enables these calculations.

Regarding the risk of disease complications, studies had to evaluate the risk of mortality/severe disease associated with ACEi and/or ARB use compared with patients not treated with these drugs, both from a population perspective or among population infected with SARS-CoV-2. ACEi or ARBs had to be reported by the investigators as a group (ACEi/ARB) or individually. We accepted controls treated with other antihypertensive drugs or without any antihypertensive drug.

In case-control studies, cases were patients with COVID-19 infection (positive test) irrespective of disease severity, and controls were matched individuals without the referred outcomes. Data about ACEi and/or ARB risks should be available.

The outcomes of interest were:

1) COVID-19 infection documented by nasophaygeal or oropharyngeal swab tests or reported by authors as having COVID-19;
2) All-cause Mortality;
3) Severe/Critical Disease according with the World Health Organization and Chinese Centre of Disease Control [6], [7].

Whenever possible, if adequate, adjusted measures were retrieved particularly for observational studies, giving preference to propensity score matching or weighting.

### 2.2 Search methods for study identification

The reviewers performed an electronic database search through MEDLINE, CENTRAL, PsycINFO and Web of Science Core Collection databases for relevant studies (Search strategy at Supplementary Table 1). The database medRxiv was also searched for unpublished pre-print manuscripts for an exploratory analysis. Relevant reviews obtained in the searching process as well as the references of potentially included studies were analysed in order to search for potential eligible studies. There were no restrictions on language or publication date. The search lastly performed at 8th June 2020.

### 2.3 Study selection and data collection process

The title and abstract screening phase of records yielded by the search was performed independently by clusters of 2 reviewers. Disagreements were resolved through consensus or by a third reviewer (DC). The studies that were not excluded went to the full-text assessment phase.

The reasons for exclusion were recorded at this stage.

The reviewers extracted study data following a pre-established data collection form. When studies presented different estimates of the outcome of interest, we extracted the most precise or adjusted measures.

Risk of bias was independently evaluated by three authors (DC, MA, ANF) using the Cochrane Risk of Bias Tool for randomized controlled trials and ROBINS-I tool for observational studies [8], [9]. The studies were qualitatively classified as at critical, serious, moderate, or low risk of bias. Risk of bias graphs were derived from these tools.

### 2.4 Statistical analysis and pooled data evaluation

We used Review Manager for statistical analysis and to derive forest plots. We used the inverse variance method and random-effects model to pool data. We reported pooled dichotomous data using odds ratios (OR) with their 95% confidence intervals (95% CIs). Heterogeneity was assessed using I^2^ [10]. We present effect estimates as OR because relative estimates are more similar across studies with different designs, populations and lengths of follow-up than absolute effects [11]. We used the hazard ratio (HR) when OR was not available nor possible to calculate. Publication bias assessment was performed through funnel plot examination and Egger test providing that a sufficient number of studies were included [12].

Exploratory analyses were performed with adjusted estimates, and only those with data of hypertensive patients. We further performed an additional exploratory analysis including unpublished (Preprint) studies found in medRxiv.

We used the Grading of Recommendations, Assessment, and Evaluation (GRADE) framework to report the overall quality of evidence. The certainty in the evidence for each outcome was graded as high, moderate, low, or very low [13].

## 3 Results

### 3.1 Included studies

**Fig. 1:**
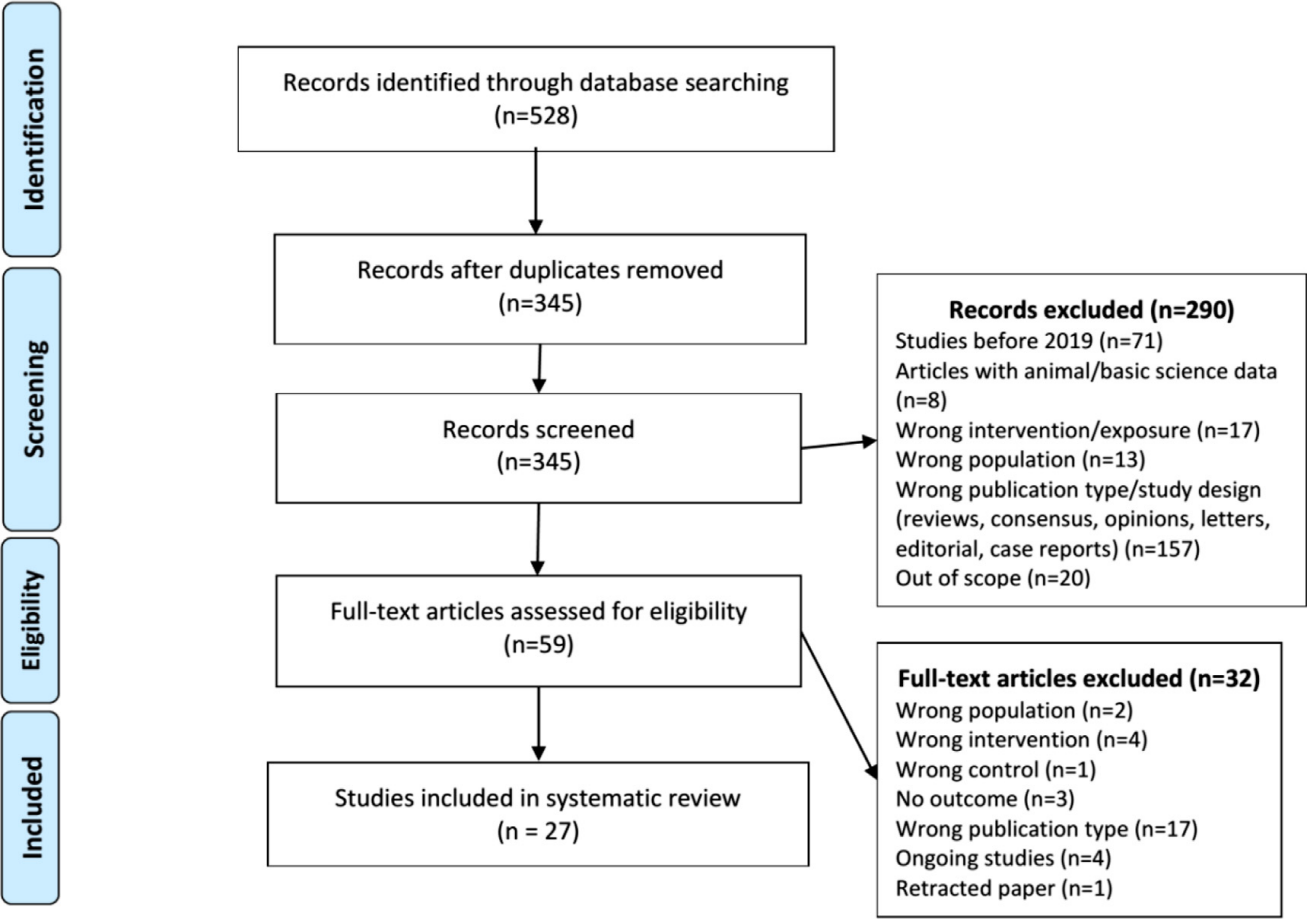
Flowchart of studies selection process.

The search returned 528 records, resulting in 27 study records after the deduplication, title and abstract screening and full-text screening (Fig. 1; details of excluded studies at Supplementary Table 2) [14], [15], [16], [17], [18], [19], [20], [21], [22], [23], [24], [25], [26], [27], [28], [29], [30], [31], [32], [33], [34], [35], [36], [37], [38], [39], [40]. There was one randomized controlled trial (a non-prespecified interim analysis of an open-label trial), 4 case-control studies (two of them – Gnavi et al – were reported in the same article) and the remaining were cohort/nested case-control studies.

The main characteristics of the included studies are depicted in Table 1. The median sample size was 522 [interquartile range 113–4051] and overall, there were 119,656 participants evaluated.

**Table 1:** Main characteristics of included studies.

Study Year	Design	Region	Population	Total/ ACEi/ARB	Control	Mean-median age / % female	Comorbidities	Outcome adjustments
RANDOMIZED CONTROLLED TRIAL								
Amat-Santos 2020	Non-planned interim analysis of an open-label RCT	Spain	Patients with aortic stenosis successfully treated with transcatheter aortic valve replacement	ACEi (ramipril): 52	Placebo: 50	83 47%	HTN: 54% CAD: 26% DM: 20% CKD: 33%	–

CASE-CONTROL STUDIES								
de Abajo 2020	Case-control study	Madrid, Spain	Case: ≥ 18 years with PCR-confirmed COVID-19 requiring hospital admission (n = 1139) Control: from database Investigación Farmacoepidemiológica en Atención Primaria (BIFAP), a Spanish primary health-care database (n = 11390) Matching 1:10 by sex and age	COVID-19 positive Total: 497 ACEi: 240 ARB: 244 Aldosterone antagonists: 38 Renin inhibitors: 1	COVID-19 negative Total: 3822 ACEi: 2192 ARB: 1616 Aldosterone antagonists:218 Renin inhibitors: 8	69 61%	HTN: 54% COVID-19+ 50% COVID-19 - CAD: 11% COVID-19+ 8% COVID-19 - DM: 27% COVID-19+ 20% COVID-19 - CKD: 8% COVID-19+ 5% COVID-19 - HF: 7% COVID-19+ 4% COVID-19 -	Age, sex, diabetes, dyslipidemia, ischemic heart disease, heart failure, atrial fibrillation, thromboembolic disease, cerebrovascular accident, chronic obstructive pulmonary disease, asthma, cancer, and chronic kidney disease
Gnavi 2020	Nested case-control study in 2 cohorts	Piedmont, Italy	Case: Discharged patients with confirmed COVID-19 infection (RT-PCR) in - Hypertensive patients (N = 316) - Cardiovasular disease* patients (N = 171) Control: Discharged patients without COVID-19 infection Matching 1:5 by sex and age	COVID-19 positive Total: 215 ACEi: NR ARB: NR Total: 93 ACEi: NR ARB: NR	COVID-19 negative Total: 1153 ACEi: NR ARB: NR Total: 475 ACEi: NR ARB: NR	71 (hypertension cohort); 75 (cardiovascular disease cohort) 31% (hypertension cohort); 22% (cardiovascular disease cohort)	HTN: 100%	Age, sex, and disease type (hypertension or cardiovascular disease)
Mancia 2020	Population-based case–control study	Lombardy Italy	Case: Positive COVID-19 patients (≥40 years old) N = 6272 Control: beneficiaries of the Regional Health Service N = 30759 Matched 1:5 by sex, age at index date, and municipality of residence	COVID-19 positive Total: 2896 ACEi: 1502 ARB: 1394	COVID-19 negative Total: 27863 ACEi: 6569 ARB: 5910	68 37%	NR	Cardiovascular disease, respiratory disease, kidney disease, cancer, antihypertensive agents, lipid lowering agents, oral hypoglycemic agents, insulin, antiplatelet agents, antiarrhythmic agents, anticoagulant agents, digitalis, nitrates, inhaled glucocorticoids, nonsteroidal antiinflamatory drugs, immunosuppressive agents, beta agonists, other drugs for respiratory disease
COHORT/NESTED CASE-CONTROL STUDIES								
Argenziano 2020	Single-center retrospective cohort study	New York, USA	Patients with hypertension and diabetes admitted in the emergency department or in the hospital for COVID-19 infection N = 1000	Total: 284 ACEI: NR ARB: NR	Non-ACEi/ARB: 716	63 40%	HTN: 60% CAD: 13% DM: 37% CKD: 14% HF: 10%	–
Bean 2020	Retrospective cohort study	London, UK	Adult COVID 19 symptomatic patients N = 1200	Total: 399 ACEi: 260 ARB: 147	Non-ACEi/ARB: 801	68 43%	HTN: 54% DM: 35% CKD: 17% HF: 9%	Age, sex, hypertension, diabetes mellitus, chronic kidney disease, ischaemic heart disease, heart failure
Chen 2020	Retrospective cohort study	Wuhan, China	Patients with hypertension and diabetes admitted in the hospital for symptomatic COVID-19 infection N = 71	Total: 31 ACEi: NR ARB: NR	Non-ACEi/ARB: 39	67 NR	HTN: 100% DM: 100%	–
Chodik 2020	Cross sectional Cohort	Tel Aviv, Israel	individuals tested for SARS-COV-2 (RT-PCR) N = 1317 positive N = 13203 negative	Total: 991 ARB 603 ACEI 388	Non-ACEi/ARB: 13,529	41 COVID-19+ 37 COVID-19– 40% COVID-19 + / 46% COVID-19 -	HTN: 14% COVID-19+ 11% COVID-19 - DM: 9% COVID-19+ 5% COVID-19 - CKD: 8% COVID-19+ 6% COVID-19 - HF: 0.2% COVID-19+ 0.6% COVID-19 -	Age, sex, SES, BMI, and co-morbidities
Yan, 2020	Multicentre retrospective case-control study	Zhejiang, China	Case: Consecutive patients presenting to hospital with confirmed diagnosis of Covid-19 infection N = 610 Control: Population-based control group N = 48667	COVID-19+ Total: 58 ACEi: 5 ARB: 53	COVID.19 - Total: 8040 ACEi: 555 ARB: 7485	49 49%	HTN: 22% DM: 10% CVD/cerebrovascular disease: 3%	Age, sex, BMI
Felice 2020	Single-centre retrospective cohort study	Treviso, Italy	Symptomatic COVID-19 hypertensive patients presenting to the emergency department N = 133	Total: 82 ACEI: 40 ARB: 42	Non-ACEi/ARB: 51	73 35%	HTN: 100% DM: 26% HF: 24%	–
Feng 2020	Multi-center retrospective cohort study	Wuhan, Shangai, Tongling China	Subgroup of hypertensive COVID 19 symptomatic patients admitted in 3 hospitals N = 113	Total: 33 ACEi: NR ARB: NR	Non-ACEi/ARB: 62	53 45%	HTN: 100%	–
Gao 2020	Single-centre retrospective cohort study	Wuhan, China	Subgroup of hypertensive COVID 19 symptomatic patients admitted in the hospital N = 710	Total: 183 ACEi: NR ARB: NR	Non-ACEi/ARB: 527	64 48%	HTN: 100% DM: 28% HF: 3% CKD: 2% MI: 1%	Propensity-matched score for mortality Age, sex, medical history of diabetes, insulin-treated diabetes, myocardial infarction, PCI/CABG, renal failure, stroke, heart failure, and COPD
Hu 2020	Retrospective single-centre cohort	Zhejiang, China	Subgroup of hypertensive COVID 19 symptomatic patients admitted in the hospital N = 149	Total: 65 ACEi: NR ARB: NR	Non-ACEi/ARB: 84	57 41%	HTN 100% DM: 20% CKD: 4%	–
Huang 2020	Retrospective single-centre cohort	Wuhan, China	Hypertensive COVID 19 symptomatic patients admitted in the hospital N = 50	Total: 20 ACEi: NR ARB: NR	Non-ACEi/ARB: 30	62 45%	HTN: 100% DM: 8% CAD: 2%	–
Imam 2020	Retrospective multicentre cohort	Detroit, USA	COVID-19 symptomatic patients N = 1305	Total: 565 ACEi: NR ARB: NR	Non-ACEi/ARB: 740	61 46%	HTN: 56% DM: 30% HF: 6% Vascular Disease: 16%	Age, comorbidities, NSAID, ACEi/ARB
Jung 2020	Cohort study	Seoul, Korea	Adult COVID 19 patients N = 5179	Total: 762 ACEI: 32 ARB: 730	Non-ACEi/ARB: 1577	45 56%	HTN: 22% DM: 17% HF: 4% CAD: 1% CKD: 5%	Age, sex, Charlson Comorbidity Index, immunosuppression, and hospital type.
Li 2020	Retrospective, single-center cohort	Wuhan, China	Hypertensive COVID 19 symptomatic hospitalized patients N = 362	Total: 115 ACEi: NR ARB: NR	Non-ACEI/ARB: 247	66 41-51%	HTN: 100% DM 35% Cerebrovascular disease: 23% CHD: 18% HF 3%	–
Mehra, 2020	Cohort/Nested case-control	169 hospitals in Asia, Europe, and North America	Hospitalized patients from Surgical Outcomes Collaborative (Surgisphere), an international registry N = 8910	Total: 1326 ACEi: 770 ARB: 556	Non-ACEi/ARB: 7584	49 40%	HTN: 26% CAD: 11% DM: 14% HF: 2% Dyslipidemia: 31% COPD: 3%	Age, sex, hypertension
Mehta, 2020	Retrospective cohort study	Ohio and Florida, USA	Patients tested for COVID-19 N = 18472 N positive = 1735 N negative = 16737	Total: 2285 ACEi: 1322 ARB: 982	Non-ACEi/ARB: 16187	49 60%	HTN: 93% DM: 46% CAD: 22% HF: 17% COPD: 14%	Propensity score: Age, sex, and presence of hypertension, diabetes, coronary artery disease, heart failure, and COPD
Meng, 2020	Retrospective single center case control	Shenzhen, China	Hospitalized patients with COVID-19 and receiving anti-hypertensive therapy N = 42	Total: 17 ACEi: NR ARB: NR	Non-ACEi/ARB: 25	65 43%	HTN: 100% DM: 24% ACEi/ARB 8% Non-ACEi/ARB CHD: 35% ACEi/ARB 8% Non-ACEi/ARB	–
Million, 2020	Retrospective cohort study	Marseille, France	COVID-19 positive tested patients N = 1061	ARB: 40	Non-ARB: 1021	44 54%	HTN: 14% DM: 7% CAD: 4% Obesity: 6% Chronic Respiratory Disease: 11%	Age, comedications, COVID-19 severity score
Montastruc 2020	Retrospective cohort study	Toulouse, France	Adult patients positive for COVID-19 admitted in the intensive care unit N = 96	Total: 35 ACEI: 12 ARB: 23	Non-ACEi/ARB: 61	63 21%	HTN: 45% DM: 28% CKD: 10% Arrythmia: 6%	–
Peng 2020	Retrospective cohort study	Wuhan, China	Hospitalized COVID-19 patients with Cardiovascular disease N = 112	Total: 22 ACEi: NR ARB: NR	Non-ACEi/ARB: 90	61 53%	DM: 33% HTN: 82%	–
Reynolds 2020	Retrospective cohort study	New York, USA	Patients tested for COVID-19 N = 12594 N positive = 5894 N negative = 6700	Risk of infection (PSM): Total 1909 ACEi: 1137 ARB: 1044 Risk of severe infection (PSM): Total 1110 ACEi: 627 ARB: 664	Risk of infection (PSM): Non-ACEi/ARB: 4344 Risk of severe infection (PSM): Non-ACEi/ARB: 2453	49 59%	HTN: 35% DM: 18% HF: 6% MI: 4% CKD: 18% COPD: 15% Current smoker: 5% Former smoker: 18%	Age; sex; race; ethnic group; body-mass index; smoking history; history of hypertension, myocardial infarction, heart failure, diabetes, chronic kidney disease, and obstructive lung disease (e.g., asthma and obstructive pulmonary diseases)
Tan, 2020	Retrospective cohort study	Wuhan, China	Subgroup of symptomatic COVID-19 patients with hypertension N = 100	Total: 31 ACEi: NR ARB: NR	Non-ACEi/ARB: 69	67 NR	HTN: 100% DM: 26% ACEi/ARB 29% Non-ACEi/ARB GI: 19% ACEi/ARB 25% Non-ACEi/ARB CKD: 7% ACEi/ARB 13% Non-ACEi/ARB CHD: 10% ACEi/ARB 16% Non-ACEi/ARB COPD: 7% ACEi/ARB 10% Non-ACEi/ARB Tumor: 3% ACEi/ARB 6% Non-ACEi/ARB	–
Tedeschi, 2020	Prospective cohort study	Bologna, Italy	Hypertensive adult COVID 19 patients hospitalized N = 311	Total: 175 ACEI: 99 ARB: 76	Non-ACEi/ARB: 136	76 28%	HTN: 100% CAD: 42% DM: 24%	Age, gender, presence of CV comorbidities and COPD
Yang, 2020	Retrospective, single-center study	Wuhan, China	Subgroup of hypertensive patients with COVID-19 hospitalized N = 126	Total: 43 ACEi: NR ARB: NR	Non-ACEi/ARB: 83	Median 67 51%	HTN: 100% DM: 30% Respiratory disease: 5% Kidney disease: 3% Hepatic disease: 6% Cardiopathy: 18% Neurological disease: 8%	–
Zhang, 2020	Retrospective cohort/nested case-control	Hubei, China	Hypertensive patients with COVID-19 hospitalized N = 522 matching 1:2	Total: 174 ACEi: NR ARB: NR	Non-ACEi/ARB: 348	median 64 47%	HTN: 100% DM: 24% CAD: 12% CKD 3% CVD: 3% COPD: 1%	Age, gender, fever, cough, dyspnea, comorbidities (diabetes, coronary heart disease, and chronic renal disease), CT-diagnosed bilateral lung lesions, and incidence of increased CRP and creatinine.
Zhou 2020	Retrospective, single-center study	Wuhan, China	Subgroup of hypertensive patients with symptomatic COVID-19 hospitalized N = 36	Total: 15 ACEi: NR ARB: NR	Non-ACEi/ARB: 21	65 47%	HTN:100% DM: 25% CVD: 19%	Age, sex, hospitalization time, time from onset to hospital admission, and whether to take ACEi or ARB

UNPUBLISHED								
Rossi 2020	Population-based prospective cohort study on archive data	Reggio Emilia, Italy	COVID-19 symptomatic patients N = 2653	Total: 818 ACEi: 450 ARB: 368	Non-ACEi/ARB: 1835	63 50%	HTN: 18% DM: 12% MI: 7% HF: 6% CKD: 3% COPD: 5% Vascular disease: 3%	Age, sex and analysis restricted to subjects with ischemic heart disease, hypertension, or heart failure
Ip 2020	Retrospective, Cohort, multicenter study	New Jersey, USA	Subgroup of hypertensive COVID 19 symptomatic hospitalized patients N = 1584	Total: 1231 ACEi: 688 ARB: 543	Non-ACEi/ARB: NR	NR	HTN: 100%	–
Liu 2020	Multicentre retrospective cohort study	Shenzhen, Wuhan, and Beijing, China	Hypertensive COVID 19 symptomatic hospitalized elderly pts (greater than65 years-old) N = 78	Total: 12 ACEi: 2 ARB: 10	Non-ACEi/ARB: 8	NR	HTN 100%	Gender
Rentsch 2020	Retrospective cohort study	Connecticut, USA	Patients from National Veterans Affairs Healthcare System tested for COVID-19 N = 3789	Total: 1532 ACEi: NR ARB: NR	Non-ACEi/ARB: 2257	66 10%	HTN: 65% DM: 38% Vascular disease: 29% COPD: 26% Alcohol use disorder: 14%	Age, sex, race, medication, residence type, comorbidities
Zeng 2020	Retrospective, single-center study	Wuhan, China	Subgroup of hypertensive patients with clinically confirmed COVID-19 hospitalized N = 75	Total: 28 ACEi: NR ARB: NR	Non-ACEi/ARB: 47	67 53%	HTN: 100% DM: 31% CVD: 21% CKD: 5%	–
Khera 2020	Retrospective Cohort	Connecticut, USA	Hypertension patients hospitalized for COVID-19 N = 7933	Total: 4587 ACEi: 2361 ARB: 2226	Non-ACEI/ARB: 3346	Median 69 53%	HTN 100% DM: 68% MI 4% HF 14%	Propensity score: age, gender, race, insurance type, conditions that may lead to selective use of ACE inhibitors and ARBs each of the comorbidities in the Charlson Comorbidity Index, and the number of anti-hypertensive agents used for the patient

*with a diagnosis of ischaemic heart disease (ICD9CM at discharge 410–414), cerebrovascular disease (430–438), or heart failure (428), and persons registered in the regional register of persons with diabetes.

Legend: RT-CPR reverse transcriptase-polymerase chain reaction; ACEi Angiotensin-converting-enzyme inhibitors; ARB angiotensin receptor blocker; RAAS renin-angiotensin-aldosterone system; SES socioeconomic status; BMI body mass index; HTN hypertension; CAD coronary artery disease; HF heart failure; DM diabetes mellitus; CKD chronic kidney disease; MI myocardial infarction; COPD Chronic obstructive pulmonary disease; CHD chronic heart disease; PCI percutaneous coronary intervention; PSM: Propensity-score matching CABG coronary artery bypass graft; NSAID Nonsteroidal anti-inflammatory drugs; GI gastrointestinal; CT computed tomography; CRP c-reactive protein; pts patients.

### 3.2 Risk of bias

The risk of bias in the included studies was moderate for studies evaluating the risk of infection, while those assessing the infection severity/mortality were classified as serious. The only randomized controlled trial had an open-label design, a small sample size (n = 102) and was not designed to assess COVID-19 outcomes as the reported results were from a non-prespecified interim analysis. The lack of outcome adjustments for important clinical factors was the main source of risk of bias. Supplementary Table 3 details the risk of bias for each study according with the outcome. Supplementary Fig. 1 overviews the proportions of risk of bias categories.

### 3.3 Risk of COVID-19 infection (positive test) associated with ACEi/ARB

**Fig. 2:**
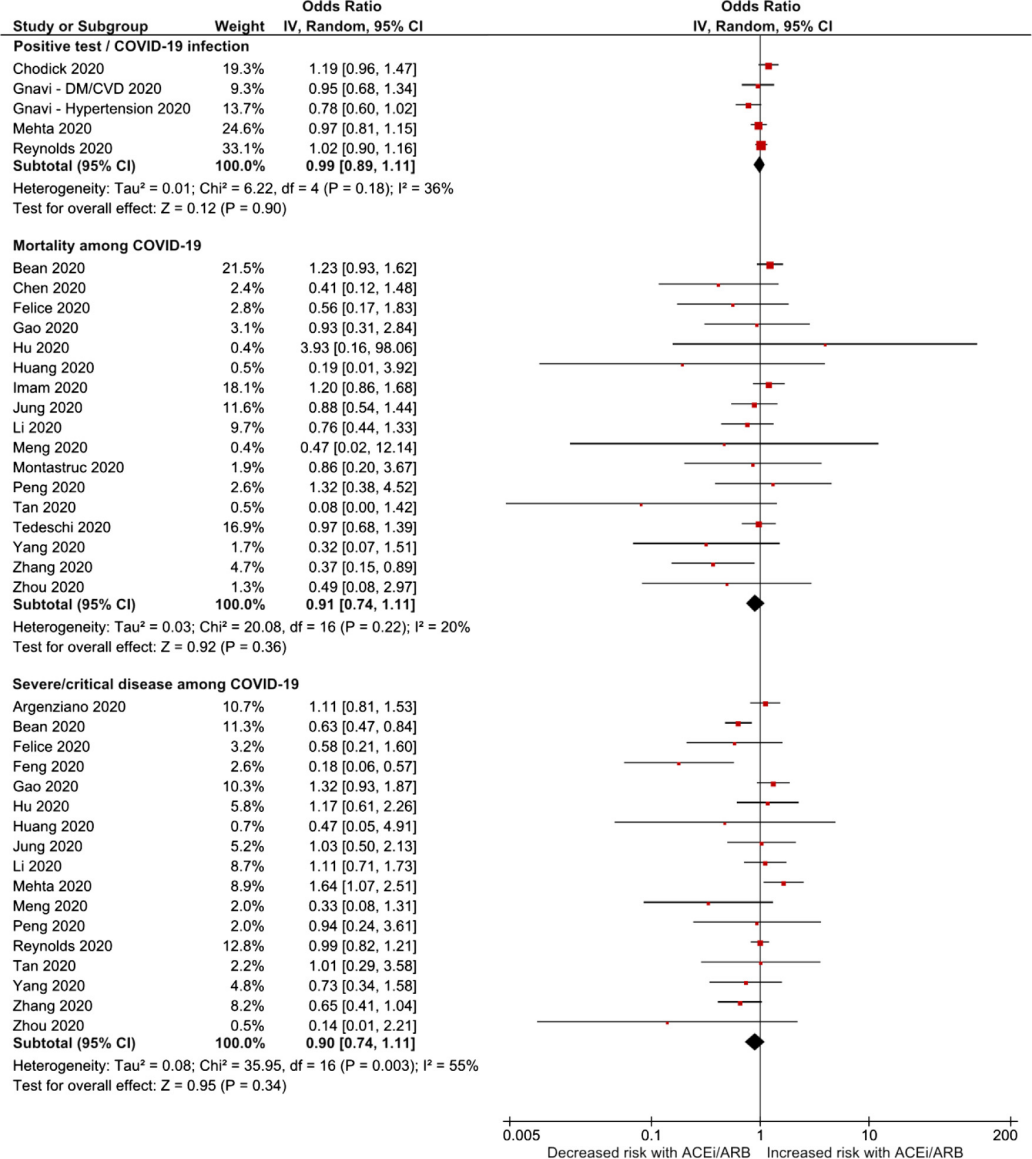
Forest plots of ACEi/ARB association with the risk of COVID-19 infection and disease severity.

Six cohorts had information about COVID-19 infection (positive test) and ACEi and/or ARB. In the meta-analysis the ACEi/ARB group was not associated with increased risk of having a positive test for COVID-19 infection (OR 0.99, 95%CI 0.91–1.11; I^2^ = 36%; 6 studies; Fig. 2), nor ACEi (OR 0.94, 95%CI 0.87–1.02; I^2^ = 0%; 7 studies) or ARB (OR 1.01, 95%CI 0.93–1.10; I^2^ = 11%; 6 studies) (Supplementary Figs. 2 and 3), individually.

### 3.4 Mortality risk associated with ACEi/ARB among patients with COVID-19 infection

**Fig. 3:**
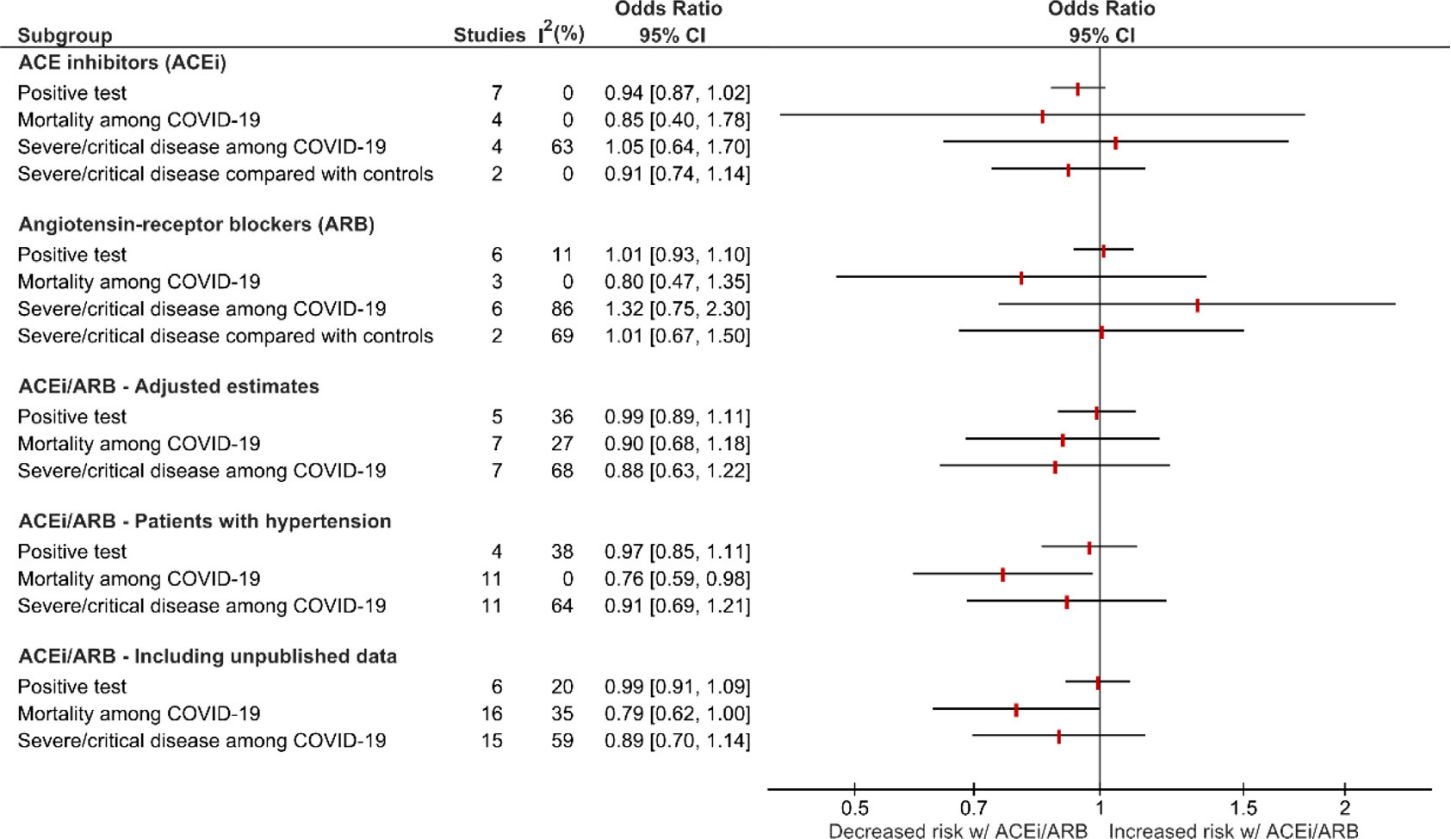
Forest plots of ACEi or ARB association with the risk of COVID-19 infection and disease severity, and the results of subanalyses of ACEi/ARB.

Regarding all-cause mortality, ACEi or ARB were associated with neither an increased nor reduction in the risk this outcome: ACEi/ARB, OR 0.91, 95%CI 0.74–1.11, I^2^ = 20%, 17 studies, Fig. 2); ACEi, OR 0.85, 95%CI 0.40–1.78, I^2^ = 0%, 4 studies; and ARB OR 0.80, 95%CI 0.47–1.35, I^2^ = 0%, 3 studies (Fig. 3; Supplementary Figs. 2 and 3).

### 3.5 Risk of severe disease associated with ACEi/ARB among patients with COVID-19 infection

The risk of severe COVID-19 disease associated with ACEi/ARB (OR 0.90, 95%CI 0.74–1.11; I^2^ = 55%; 17 studies; Fig. 2), ACEi (OR 1.05, 95%CI 0.64–1.70; I^2^ = 63%; 4 studies) or ARB (OR 1.32, 95%CI 0.75–2.30; I^2^ = 86%; 6 studies) individually was not significantly increased nor decreased (Fig. 3; Supplementary Figs. 2 and 3).

### 3.6 Risk of severe disease associated with ACEi/ARB compared with populational controls

Two case-control studies evaluated the risk of severe COVID-19 associated with ACEi/ARB using populational controls as reference [17], [27]. One only study had data about a grouped estimate of ACEi/ARB and the results did not support the hypothesis that ACEi/ARB was associated with severe COVID-19 (OR 1.08, 95%CI 0.79–1.47; 1 study) [27]. Two studies supplied data for ACEi and ARB individually [17], [27], and the pooled estimates for both evaluations showed no significant effects (ACEi: OR 0.91, 95% 0.72–1-14; I^2^ = 0%, 2 studies; ARB: 1.01, 95%CI 0.67–1.50; I^2^ = 69%; 2 studies; Fig. 3; Supplementary Figs. 2 and 3).

### 3.7 Publication bias risk assessment

We performed the Egger test in the evaluations of ACEi/ARB with more than 10 studies to determine whether publication bias exists. The Egger test was not statistically significant in the risk of having COVID-19 infection (p-value 0.64), risk of mortality among those symptomatic COVID-19 (p-value 0.09), and risk of severe disease among those with COVID (p-value 0.42). The funnel plots are depicted in Supplementary Figure 4.

### 3.8 Sub-analyses

We performed sub-analyses of ACEi/ARB association including only studies with adjusted estimates, hypertensive patients, and including unpublished data (Fig. 3).

The analysis of studies with adjusted estimates did not find any significant association between ACEi/ARB and risk of infection (OR 0.99, 95%CI 0.89–1.11, I^2^ = 35%, 5 studies), mortality (OR 0.90, 95%CI 0.68–1.18, I^2^ = 27%) and severe/critical disease (OR 0.88, 95%CI 0.63–1.22, I^2^ = 68%) among patients with COVID-19 (Fig. 3, Supplementary Figure 5).

Analysing only the data from hypertensive patients, the risk of developing the infection in patients treated with ACEi/ARB was not significantly increased (OR 0.97, 95%CI 0.85–1.11; I^2^ = 38%) (Fig. 3, Supplementary Figure 6). The mortality risk (OR 0.76, 95%CI 0.59–0.98; I^2^ = 0%) was significantly decreased in this population while the risk of developing severe disease (OR 0.91, 95%CI 0.69–1.21; I^2^ = 64%) was not statistically significant (Fig. 3, Supplementary Figure 6).

Considering both published and unpublished data retrieved from 7 additional studies (supplementary Table 4), there was a non-statistically significant association between ACEi/ARB and decreased mortality risk among COVID-19 patients (OR 0.79, 95%CI 0.62–1.00, I^2^ = 0%) (Fig. 3, Supplementary Figure 5). The risk of infection (OR 0.99, 95%CI 0.91–1.09, I^2^ = 20%) and the risk of severe/critical disease (OR 0.89, 95%CI 0.70–1.14, I^2^ = 59%) were neither significantly increased nor decreased (Fig. 3, Supplementary Figure 7).

#### 3.8.1 Assessment of confidence in cumulative evidence

Table 2 presents a summary of findings table which summarizes the results obtained only for the associations found for grouped ACEi/ARB exposure, according to certainty of the evidence (GRADE). The current evidence is that ACEi/ARB use is not associated with increased clinically significant risk of having a positive test with moderate confidence. Mortality risk among COVID-19 patients was significantly decreased, but the confidence of these data was graded as low (Table 2). The confidence concerning the association of ACEi/ARB and risk severe/critical disease among COVID-19 patients was very low (Table 2).

**Table 2:** Summary of finding table with the GRADE approach.

Outcomes	№ of studies	Certainty of the evidence (GRADE) for the lack of effect*	Relative effect (95% CI)
**ACEi/ARB and COVID-19**			
Positive test	5 observational studies		**0.99** (0.89–1.11)
Mortality among COVID-19 patients	17 observational studies		**OR 0.91** (0.74–1.11)
Severe or critical disease	17 observational studies		**OR 0.90** (0.74–1.11)

*The threshold for clinically significant effect (harm) was arbitrarily established as an increase of 25% in the odds of the outcome (a measure suggested by GRADE [43]).

**CI:** Confidence interval; **OR:** Odds ratio.

## 4 Discussion

The main finding of this systematic review was that ACEi/ARB were not associated with increased risk of being infected (moderate confidence), and among patients with COVID-19 the exposure to ACEi/ARB did not increase the risk of severe disease (very low confidence) or mortality (low confidence). In our exploratory analysis that only included hypertensive patients, ACEi/ARB were associated with a decreased mortality risk among COVID-19 patients however the data quality/risk of bias and the fragility of this exploratory analysis precludes definite and robust conclusions about the potential benefit. The other exploratory analyses also did not suggest harm, assuring the safety for the use of these drugs.

The rationale for this research was mainly based on the correspondence publication of Lancet Respiratory Medicine where Lei Fang and colleagues found that a significant number of patients with severe infection or death from SARS-CoV-2 were hypertensive, diabetic or had cardio-cerebrovascular disease and that these conditions are often treated with ACEi or ARB [3]. They hypothesized that the risk of infection or death might be increased in this group of patients due to an increase in the expression of ACE2 which can facilitate the entrance of SARS-CoV-2 into the cells [3]. The publication gained prominence in the scientific community and led to alarmism in the non-scientific community, given the high number of patients taking these drugs.

Given that the suspension of ACEI or ARBs can lead to decompensation of the underlying pathologies and there were no robust studies to corroborate the aforementioned hypothesis (data from only small preclinical studies), this led to some of the main scientific societies such as the American Heart Association, the American College of Cardiology, the Council on Hypertension of the European Society of Cardiology, and European Society of Hypertension, to publish recommendations to warn against discontinuing these drugs in the absence of clear clinical evidence of harm [41]. Our data are important because they validate these recommendations.

Despite ACEi and ARB having pharmacodynamic effects in the same pathway, the specific site of drug action may hypothetically lead to different effects, particularly in the risk of infectious diseases. Previous systematic review evaluating the potential role of ACEi in the prevention of pneumonia [42]. At that time the putative protective mechanism was thought to be related with enhanced cough reflex related to bradykinin and substance P, both derived from the inhibition of ACE [42]. Nowadays, the mechanisms are still speculative but hypothetically both ACEi and ARB may provide lung protection through the activation of angiotensin II-receptors type 2 (AT_2_R) and Mas receptors. The potential role of ACE2 in the case of SARS-CoV-2 infection is still ambiguous. While its increase may supply pathways for SARS-CoV-2 entrance into the cells [2], it is known that cleaved and shedded ACE2 leads to the breakdown of Angiotensin II to Angiotensin 1-7 (directly or indirectly increased with ARB or ACEi, respectively) have anti-inflammatory and anti-fibrotic effect through Mas receptors [41], [43]. The SARS-CoV-2 infection also leads to a downregulation of ACE2, that was associated with increased lung injury in animal models [44], [45]. Despite these ambiguous roles of ACE2, it is important to mention that relationship of serum/urinary ACE2 and tissue concentrations and use of ACEi/ARB is not well established, particularly in humans [46], [47], [48], and the clinical relevance of such relationships point towards a neutral effect according to our data. In order to further explore the potential role ACE2 and ACEi/ARB in the Influenza A infection, which share the same lung injury pathway as SARS-CoV-2, Chung et al analyzed the data of more than 5 million people in the UK followed for a median of 8.7 years and they found that ACEi and ARB exposure were associated with a decreased risk of Influenza A infection [49].

The data of this review are also important to reassure the safety of ACEi/ARB after the retraction of a large observational study that supported the safety of ACEi/ARB and showed a potential association of ACEi with lower COVID-19 mortality (Mehra MR et al N Eng J Med 2020). The authors asked for paper retraction after some concerns about the study and the impossibility of having a third party review on their data and analyses. Therefore, and despite the retraction, considering our data (without the retracted study), it seems reasonable to claim that ACEi/ARB are not harmful, despite the limitations reflected in the GRADE confidence. This supports the recommendations for not stopping the therapeutic use of ACEi/ARB. For potential benefit assessment, as seen in the hypertensive subgroup, further studies, such as the Elimination or prolongation of ACE inhibitors and ARB in Coronavirus Disease 2019 (REPLACECOVID) or Stopping ACE-inhibitors in COVID-19 (ACEI-COVID), Coronavirus ACEi/ARB Investigation (CORONACION) will provide more insights.

Our data are limited by the studies risk of bias which includes their observational nature for most of them. Pooling data of studies with different designs that evaluated different populations should also be considered as a potential limitation. Nevertheless, it increases the power and external validity of obtained data. In some studies, the risk of severe/critical disease was retrieved from specific outcomes such as the need of mechanical invasive ventilation or acute respiratory distress syndrome. This could explain the heterogeneity found in this outcome, but exclusion of these studies did not decrease the statistical heterogeneity and it remained substantial in the sub-analyses (data not shown). Lastly in these results only reflect the impact of ACEi and/or ARB. Other modulators of the renin-angiotensin-aldosterone system such renin inhibitors (aliskiren), mineralocorticoid receptor antagonists (spironolactone or epleronone), or even sacubitril were not evaluated in this review. In fact these drugs are residual considering the prescription of ACEi or ARB that in the de Abajo study we used the odds ratio of renin-angiotensin-aldosterone inhibitors as ACEi and ARB represented more than 90% of patients treated with the drugs of this group [27].

## 5 Conclusions

Our systematic review with meta-analysis did not suggest that the exposure to ACEi/ARB increases the risk of having the SARS-CoV-2 infection or developing severe stages of the disease, which supports the position papers of several medical associations recommending for not withholding these drugs in people already treated with them. Our data also showed a statistically significant association between ACEi/ARB exposure and reduction in COVID-19 mortality in hypertensive patients, but the frailty of the data and analysis precludes definite conclusions and emphasizes the need of further robust data.

## Declaration of Competing Interest

DC in the last 3 years has participated in educational conferences/congresses (including travel, accommodation, and/or hospitality) and has received speaker/consultant fees from Daiichi Sankyo, Menarini, Roche and Merck-Serono. FJP that has received speaker and consultant fees from Bayer, Boehringer Ingelheim, Daiichi, Sankyo and Astra Zeneca.
