# Supplementary material for: Angiotensin-converting enzyme inhibitors and angiotensin-receptor blockers and the risk of COVID-19 infection or severe disease: Systematic review and meta-analysis
Source: Int J Cardiol Heart Vasc. 2020 Aug 27;31:100627. doi: 10.1016/j.ijcha.2020.100627 (PMC7451091; doi:10.1016/j.ijcha.2020.100627)
Supplement: Supplementary data 1 [file mmc1.pdf]

SUPPLEMENTARY DATA

Angiotensin-converting enzyme inhibitors and angiotensin-receptor blockers and the risk of COVID-19 infection or severe disease: Systematic Review and meta-analysis of observational studies

Daniel Caldeira MD PhD, Mariana Alves MD, Ryan Gouveia e Melo MD, Pedro Silvério António MD, Nélson Cunha MD, Afonso Nunes-Ferreira MD, Luisa Prada MD, João Costa MD PhD, Fausto J Pinto MD PhD

Content

Supplementary Table 1 – Search Strategy 3

Supplementary Table 2: Key studies excluded at full-text appraisal stage, with reasons. 4

Supplementary Table 3 – Risk of bias for each study, *per* outcome. 5

Supplementary Table 4 – References of unpublished studies included. 6

Supplementary Figure 1 – Overview of risk of bias classification 7

Supplementary Figure 2: Forest plots of ACEi association with the risk of COVID-19 infection and disease severity. 8

Supplementary Figure 3: Forest plots of ARB association with the risk of COVID-19 infection and disease severity. 9

Supplementary Figure 4: Funnel plots for grouped ACEi/ARB evaluations and respective outcomes.. 10

Supplementary Figure 5: Forest plots of ACEi/ARB association in studies with adjusted estimates with the risk of COVID-19 infection and disease severity. 11

Supplementary Figure 6: Forest plots of ACEi/ARB association with the risk of COVID-19 infection and disease severity, in studies including only patients with hypertension. 12

Supplementary Figure 7: Forest plots of ACEi/ARB association with the risk of COVID-19 infection and disease severity, including unpublished data from medRxiv............................... 13

Supplementary Table 1 – Search Strategy

#	Searches using OVID
1	exp angiotensin-converting enzyme inhibitors/
2	(angiotensin-converting enzyme inhibitor or ACE-inhibitor* or acei or ace inhibitor or alacepril or benazepril or benazeprilat or captopril or ceranapril or cilazapril or cilazaprilat or delapril or enalapril or enalaprilat or fosinopril or fosinoprilic acid or imidapril or libenzapril or lisinopril or moexipril or omapatrilat or perindopril or perindoprilat or quinapril or quinaprilat or ramipril or ramiprilat or rentiapril or spirapril or temocapril or trandolapril or zofenopril).af.
3	exp Angiotensin Receptor Antagonists/
4	(ARB* or angiotensin?receptor?blocker or (angiotensin adj2 receptor adj2 (antagonist or blocker)) or candesartan or eprosartan or enoltasosartan or irbesartan or Losartan or olmesartan or saprisartan or tasosartan or telmisartan or valsartan).af.
5	1 or 2 or 3 or 4
6	("2019 nCoV" or 2019nCoV or "2019 novel coronavirus" or "COVID 19" or COVID19 or "new coronavirus" or "novel coronavirus" or "SARS CoV-2" or (Wuhan and coronavirus) or "COVID 19" or "SARS-CoV" or "2019-nCoV" or "SARS-CoV-2 SARS-CoV2").af.
7	5 and 6
8	remove duplicates from 7

Supplementary Table 2: Key studies excluded at full-text appraisal stage, with reasons.

Study	Reason for exclusion
**Murray 2020 Cardiovasc Res**	Wrong publication type: Background/narrative review article
**Piva 2020 J Crit Care**	Wrong outcome: no data between exposure and outcomes of interest
**Rothlin 2020 Drug Devel Res**	Wrong publication type: Background/narrative review article
**Sommerstein 2020 J Am Heart Assoc**	Wrong publication type: Background/narrative review article
**Morfeld 2020 Gesundheitswesen**	Wrong outcome: no data between exposure and outcomes of interest
**Rico-Mesa 2020 Curr Cardiol Rep**	Wrong publication type: Background/narrative review article
**Slawinski 2020 Kardiol Pol**	Wrong publication type: review article
**Speth 2020 J Travel Med**	Wrong publication type: Background article/comment
**Sriram K 2020 Clin Pharmacol Ther**	Wrong publication type: review of basic science studies about ACE
**Liu Y 2020 Sci China Life Sci.**	Wrong intervention: No data about ACEi/ARB exposure
**Ciulla 2020 Eur Heart J**	Wrong outcome: not enough data to establish the risk of exposure and outcomes of interest
**Aronson 2020 BMJ**	Wrong publication type: Editorial
**Cure 2020 Diabetes Metab Syndr.**	Wrong publication type: review of pathophysiology
**Guo 2020 Zhonghua nei ke za zhi**	Wrong control: case series of 4 patients treated with aliskiren for blood pressure control during hospitalization for severe COVID-19; no comparator
**Zangrillo 2020 Crit Care**	Wrong intervention: Angiotensin II (agonist) as treatment; also no comparator
**Vaduganathan 2020 NEJM**	Wrong publication type: review
**Lopes 2020 Am Heart J**	Wrong publication type: Study rationale/protocol
**Sama 2020 Eur Heart J**	Wrong population: patients without SARS-CoV-2 infection / COVID-19
**Di Castelnuovo 2020 Hypertension**	Wrong publication type: Study rationale
**Furuashi 2020 Hypertens Res**	Wrong publication type: Editorial
**Snyder 2020 Pharmacogenomics**	Wrong publication type: Background/narrative review article
**Canata 2020 Eur Heart J Cardiovasc Pharmacother**	Wrong intervention – continuation vs. discontinuation ACEi/ARB
**Turgeon 2020 Can J Cardiol**	Wrong publication type: Editorial
**Sunden-Cullberg 2020 Hypertens**	Wrong population: non-COVID
**De Vries 2020 Neth Heart J**	Wrong publication type: review
**Bobkova 2020 Med Hypotheses**	Wrong publication type: review
**Chen 2020 J Med Virol**	Wrong intervention: Arbidol (hemagglutinin)
**Mehra 2020 New Eng J Med**	Retracted paper
**NCT04353596**	Ongoing trial - ARB/ACEi
**NCT04311177**	Ongoing trial - ARB
**NCT04318418**	Ongoing case-control study; same as Di Castelnuovo [Hypertension]
**NCT04312009**	Ongoing trial - ARB

Supplementary Table 3 – Risk of bias for each study, *per* outcome.

STUDY (FIRST AUTHOR SURNAME) / OUTCOME	RISK OF INFECTION	RISK OF MORTALITY	RISK OF SEVERE DISEASE
**AMAT-SANTOS**	Moderate*	Serious*	-
**ARGENZIANO**	-	-	Serious
**BEAN**	-	Serios	Serious
**CHODICK**	Moderate	-	-
**CHEN**	-	Serious	-
**DE ABAJO**	Moderate	-	Moderate
**FELICE**		Serious	Serious
**FENG**	-	-	Serious
**GAO**		Moderate	Serious
**GNAVI**	Serious	-	-
**HU**		Serious	Serious
**HUANG**	-	Serious	Serious
**IMAM**			
**JUNG**	-	Moderate	Moderate
**LI**	-	Serious	Serious
**MANCIA**	Moderate	Moderate	-
**MEHRA**	-	Serious	-
**MEHTA**	Moderate	-	Moderate
**MENG**	-	Serious	Serious
**MILLION**	-	Serious	-
**MONTASTRUC**	-	Critical	-
**PENG**	-	Serious	Serious
**REYNOLDS**	Moderate	-	Moderate
**TAN**	-	Serious	Serious
**TEDESCHI**	-	Serious	-
**YANG**	-	Serious	Serious
**ZHANG**	-	Serious	Serious
**ZHOU**	-	Serious	Serious
**UNPUBLISHED**			
**IP****	-	Critical	-
**KHERA****	-	Critical	-
**LIU****	-	-	Critical
**RENTSCH****	Critical	-	Critical
**ROSSI****	-	Critical	-
**YAN ****	Critical	-	Critical
**ZENG****	-	Critical	-

* Using the Cochrane Risk of Bias Tool; The remaining studies were evaluated with ROBINS-I.

**Unpublished studies were classified by the reviewers as having critical risk of bias due to this status.

Supplementary Table 4 – References of unpublished studies included.

References of unpublished studies
Rossi PG, Marino M, Formisano D, Venturelli F, Vicentini M, Grilli R, et al. Characteristics and outcomes of a cohort of SARS-CoV-2 patients in the Province of Reggio Emilia, Italy. medRxiv 2020
Ip A, Parikh K, Parrillo JE, Mathura S, Hansen E, Sawczuk IS, et al. Hypertension and Renin-Angiotensin-Aldosterone System Inhibitors in Patients with Covid-19. medRxiv 2020
Liu Y, Huang F, Xu J, Yang P, Qin Y, Cao M, et al. Anti-hypertensive Angiotensin II receptor blockers associated to mitigation of disease severity in elderly COVID-19 patients. medRxiv 2020
Rentsch CT, Kidwai-Khan F, Tate JP, Park LS, King JT, Skanderson M, et al. Covid-19 Testing, Hospital Admission, and Intensive Care Among 2,026,227 United States Veterans Aged 54-75 Years. medRxiv 2020
Yan H, Valdes AM, Vijay A, Wang S, Liang L, Yang S, et al. Role of Drugs Affecting the Renin-Angiotensin-Aldosterone System on Susceptibility and Severity of COVID-19: A Large Case-Control Study from Zheijang Province, China. medRxiv 2020
Zeng Z, Sha T, Zhang Y, Wu F, Hu H, Li H, et al. Hypertension in patients hospitalized with COVID-19 in Wuhan, China: A single-center retrospective observational study. medRxiv 2020
Khera R, Clark C, Lu Y, Guo Y, Ren S, Truax B, et al. Association of Angiotensin-Converting Enzyme Inhibitors and Angiotensin Receptor Blockers with the Risk of Hospitalization and Death in Hypertensive Patients with Coronavirus Disease-19. medRxiv 2020

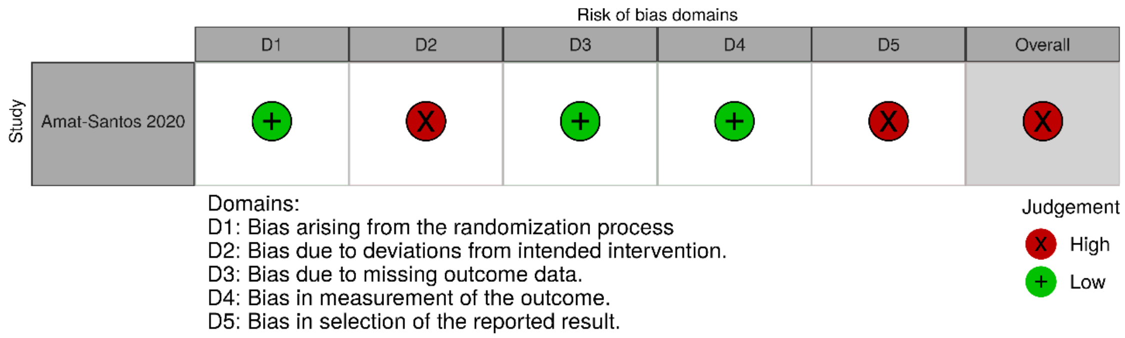

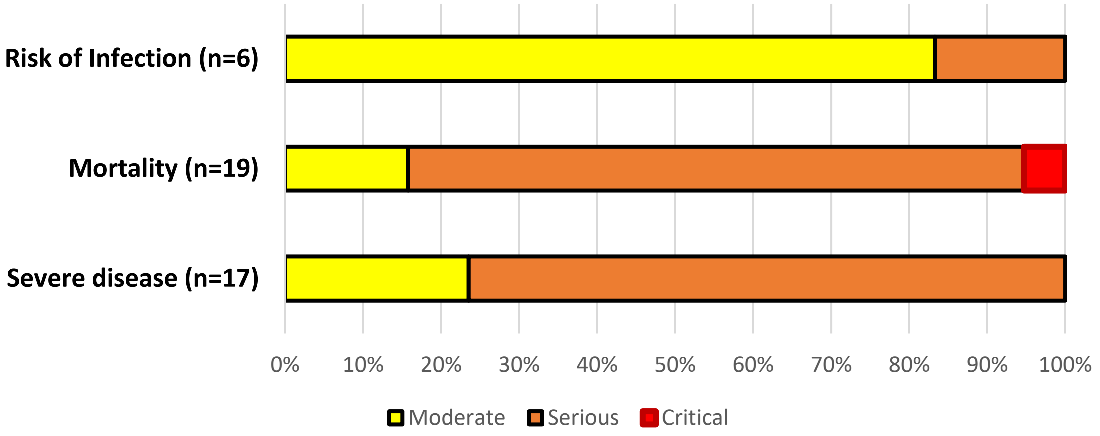

Supplementary Figure 1 – Overview of risk of bias classification; Upper figure for randomized controlled trial with risk of bias tool; and lower figure for observational studies with ROBINS-I

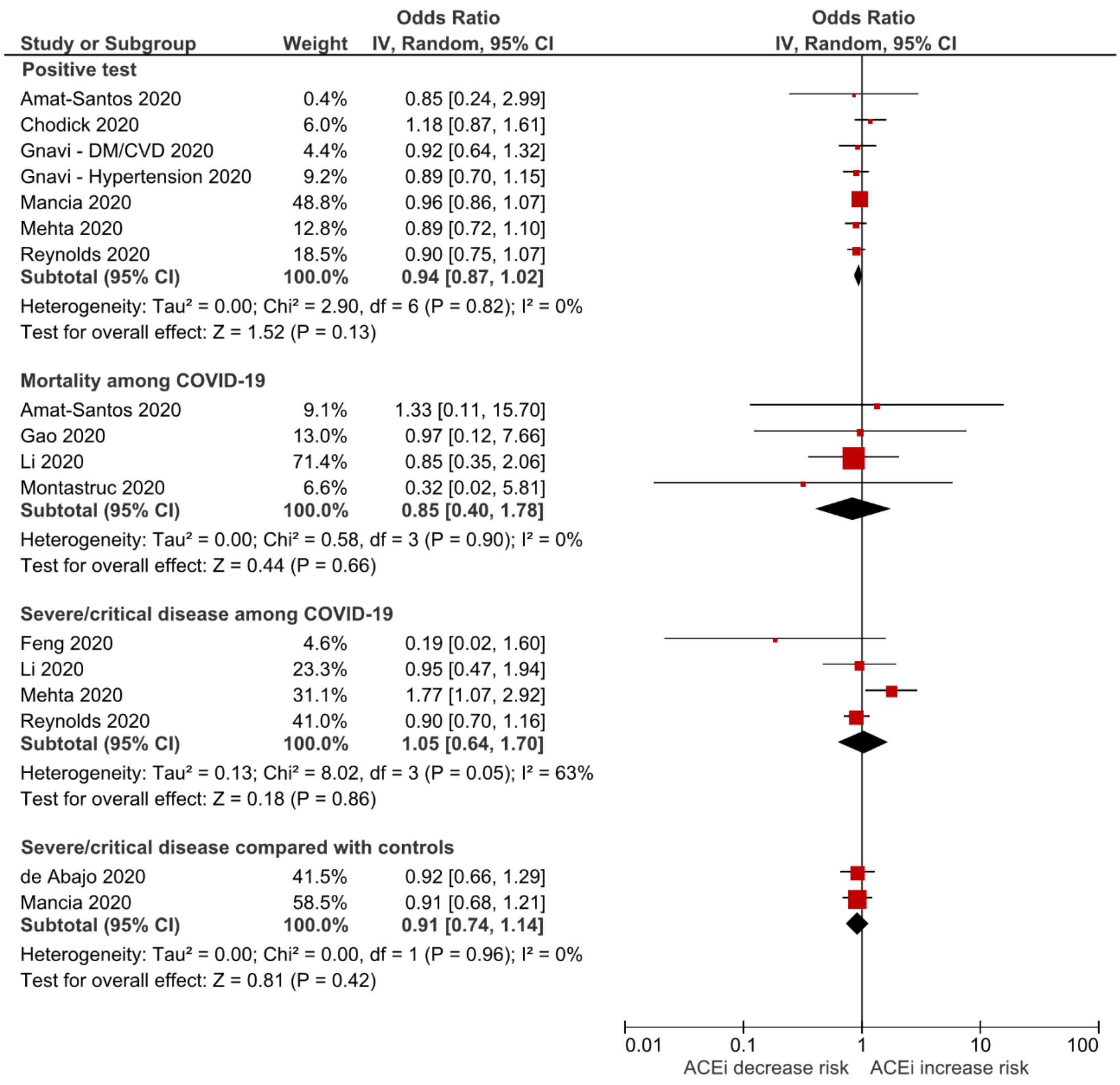

Supplementary Figure 2: Forest plots of ACEi association with the risk of COVID-19 infection and disease severity.

Study or Subgroup	Weight	Odds Ratio IV, Random, 95% CI
**Positive test**		
Chodick 2020	6.2%	1.29 [0.93, 1.79]
Gnavi - DM/CVD 2020	4.7%	1.03 [0.70, 1.50]
Gnavi - Hypertension 2020	9.8%	0.90 [0.70, 1.17]
Mancia 2020	45.8%	0.95 [0.86, 1.05]
Mehta 2020	12.2%	1.09 [0.87, 1.37]
Reynolds 2020	21.3%	1.09 [0.93, 1.29]
**Subtotal (95% CI)**	**100.0%**	**1.01 [0.93, 1.10]**
Heterogeneity: Tau² = 0.00; Chi² = 5.65, df = 5 (P = 0.34); I² = 11%		
Test for overall effect: Z = 0.31 (P = 0.76)		
**Mortality among COVID-19**		
Gao 2020	17.3%	0.96 [0.27, 3.42]
Li 2020	69.5%	0.75 [0.40, 1.42]
Montastruc 2020	13.2%	0.86 [0.20, 3.67]
**Subtotal (95% CI)**	**100.0%**	**0.80 [0.47, 1.35]**
Heterogeneity: Tau² = 0.00; Chi² = 0.12, df = 2 (P = 0.94); I² = 0%		
Test for overall effect: Z = 0.83 (P = 0.40)		
**Severe/critical disease among COVID-19**		
Feng 2020	11.1%	0.23 [0.07, 0.73]
Gao 2020	19.7%	1.30 [0.88, 1.93]
Li 2020	18.6%	1.21 [0.74, 2.00]
Mehta 2020 (1)	17.9%	1.16 [0.67, 2.02]
Million 2020	11.8%	18.00 [6.01, 53.90]
Reynolds 2020	20.9%	0.93 [0.72, 1.19]
**Subtotal (95% CI)**	**100.0%**	**1.32 [0.75, 2.30]**
Heterogeneity: Tau² = 0.37; Chi² = 34.62, df = 5 (P < 0.00001); I² = 86%		
Test for overall effect: Z = 0.97 (P = 0.33)		
**Severe/critical disease compared with controls**		
de Abajo 2020	46.8%	1.25 [0.88, 1.77]
Mancia 2020	53.2%	0.83 [0.63, 1.10]
**Subtotal (95% CI)**	**100.0%**	**1.01 [0.67, 1.50]**
Heterogeneity: Tau² = 0.06; Chi² = 3.21, df = 1 (P = 0.07); I² = 69%		
Test for overall effect: Z = 0.03 (P = 0.98)		

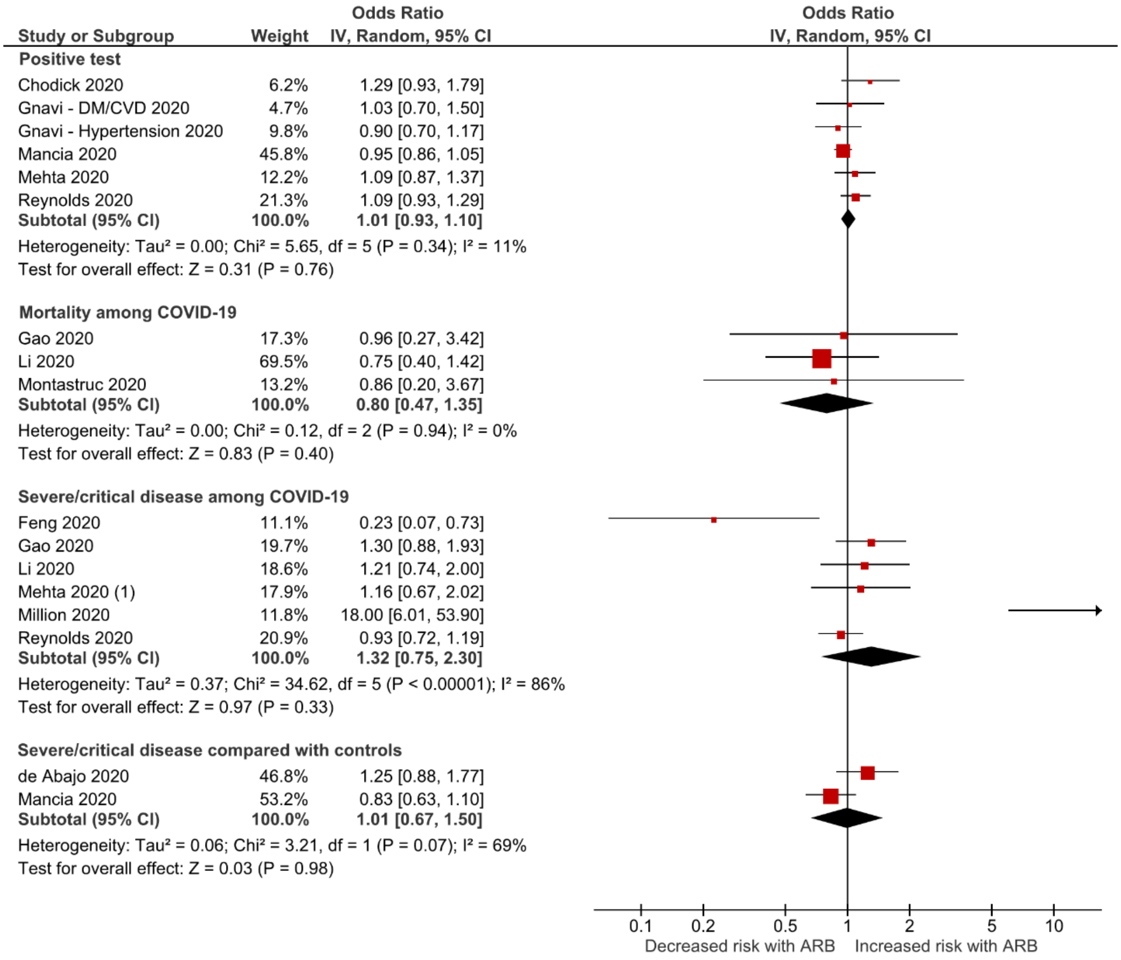

Supplementary Figure 3: Forest plots of ARB association with the risk of COVID-19 infection and disease severity.

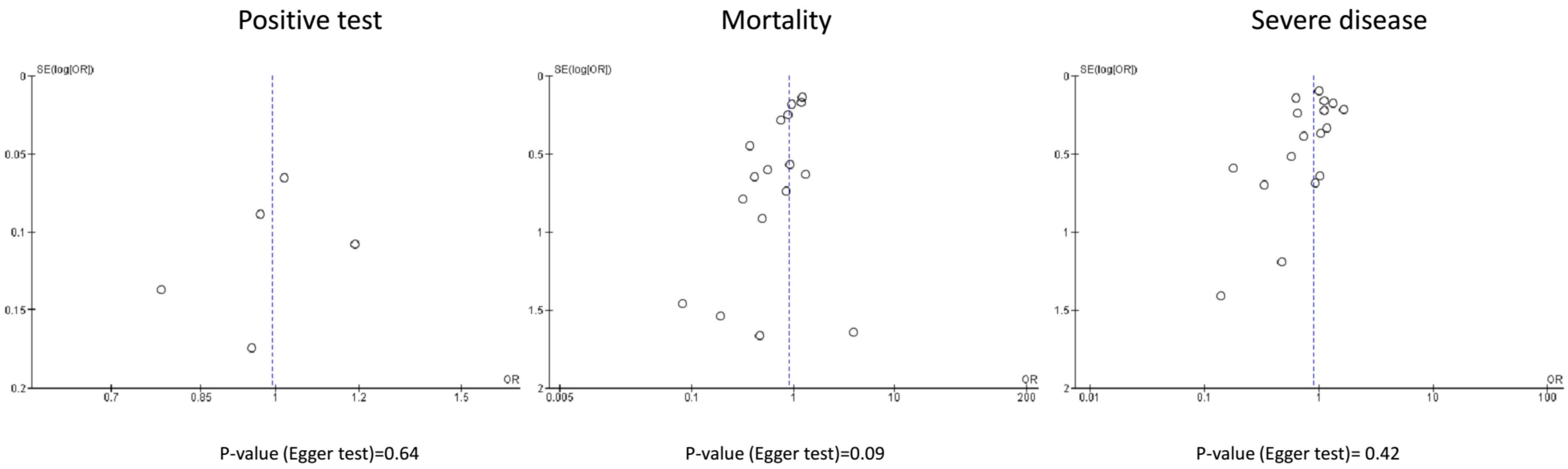

Supplementary Figure 4: Funnel plots for grouped ACEi/ARB evaluations and respective outcomes.

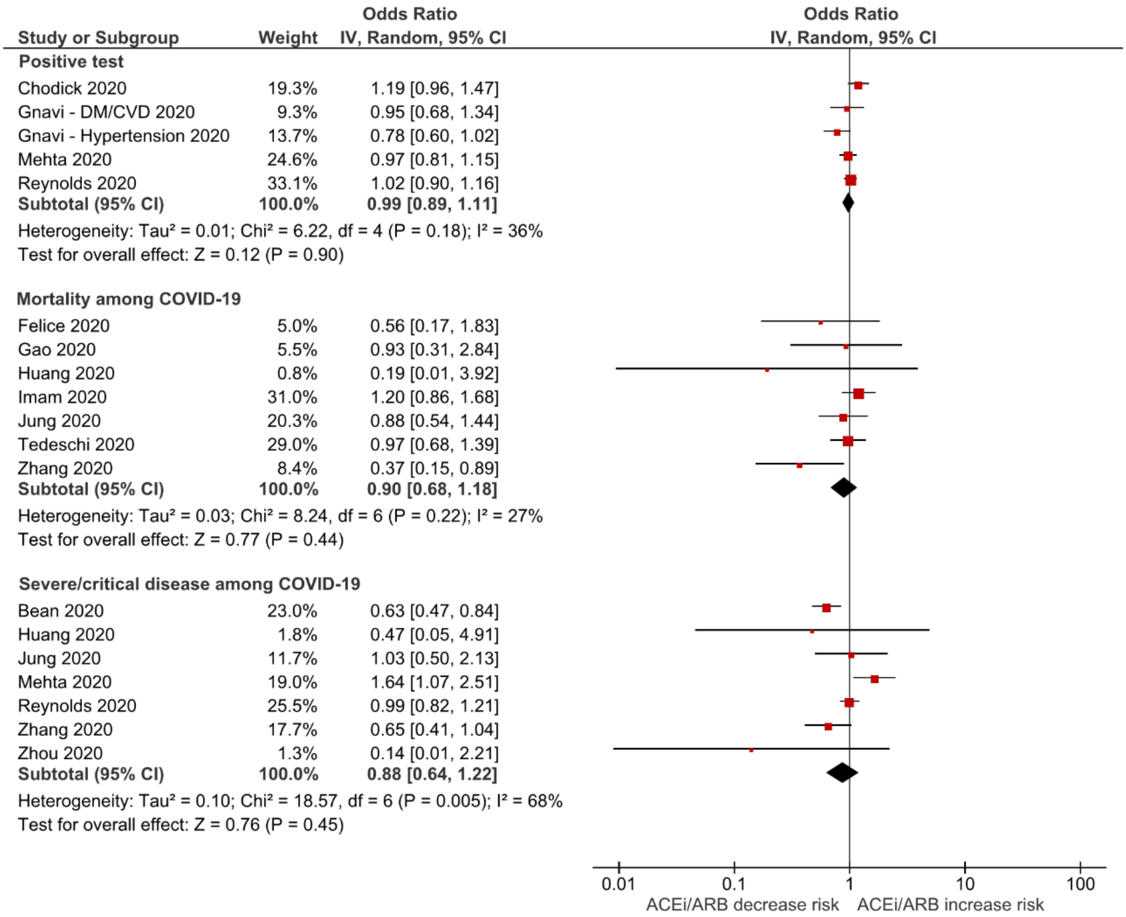

Supplementary Figure 5: Forest plots of ACEi/ARB association in studies with **adjusted estimates** with the risk of COVID-19 infection and disease severity.

Study or Subgroup	Weight	Odds Ratio IV, Random, 95% CI
**Positive test**		
Gnavi - DM/CVD 2020	12.3%	0.95 [0.68, 1.34]
Gnavi - Hypertension 2020	17.8%	0.78 [0.60, 1.02]
Mehta 2020	31.1%	0.97 [0.81, 1.15]
Reynolds 2020	38.8%	1.08 [0.95, 1.24]
**Subtotal (95% CI)**	**100.0%**	**0.97 [0.85, 1.11]**
Heterogeneity: Tau² = 0.01; Chi² = 4.84, df = 3 (P = 0.18); I² = 38%		
Test for overall effect: Z = 0.44 (P = 0.66)		
**Mortality among COVID-19**		
Chen 2020	4.0%	0.41 [0.12, 1.48]
Felice 2020	4.6%	0.56 [0.17, 1.83]
Gao 2020	5.2%	0.93 [0.31, 2.84]
Hu 2020	0.6%	3.93 [0.16, 98.06]
Huang 2020	0.7%	0.19 [0.01, 3.92]
Li 2020	20.8%	0.76 [0.44, 1.33]
Meng 2020	0.6%	0.47 [0.02, 12.14]
Tedeschi 2020	50.2%	0.97 [0.68, 1.39]
Yang 2020	2.7%	0.32 [0.07, 1.51]
Zhang 2020	8.4%	0.37 [0.15, 0.89]
Zhou 2020	2.0%	0.49 [0.08, 2.97]
**Subtotal (95% CI)**	**100.0%**	**0.76 [0.59, 0.98]**
Heterogeneity: Tau² = 0.00; Chi² = 8.92, df = 10 (P = 0.54); I² = 0%		
Test for overall effect: Z = 2.11 (P = 0.03)		
**Severe/critical disease among COVID-19**		
Feng 2020	4.4%	0.18 [0.06, 0.57]
Gao 2020	15.2%	1.32 [0.93, 1.87]
Hu 2020	9.4%	1.17 [0.61, 2.26]
Huang 2020	1.3%	0.47 [0.05, 4.91]
Li 2020	13.2%	1.11 [0.71, 1.73]
Mehta 2020	13.5%	1.64 [1.07, 2.51]
Meng 2020	3.4%	0.33 [0.08, 1.31]
Reynolds 2020	18.0%	0.97 [0.79, 1.19]
Yang 2020	7.9%	0.73 [0.34, 1.58]
Zhang 2020	12.7%	0.65 [0.41, 1.04]
Zhou 2020	1.0%	0.14 [0.01, 2.21]
**Subtotal (95% CI)**	**100.0%**	**0.91 [0.69, 1.21]**
Heterogeneity: Tau² = 0.10; Chi² = 25.29, df = 10 (P = 0.005); I² = 60%		
Test for overall effect: Z = 0.63 (P = 0.53)		

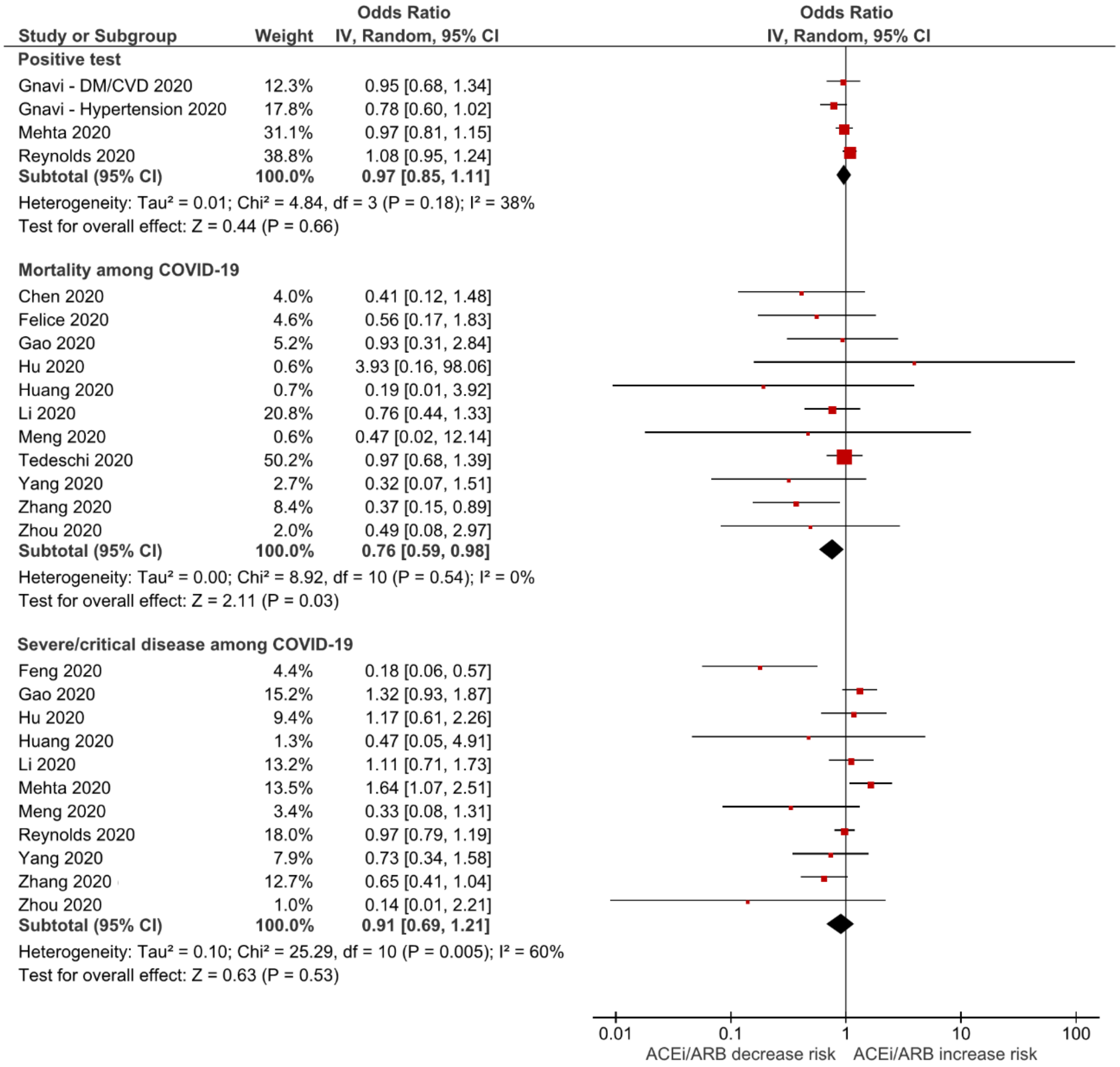

Supplementary Figure 6: Forest plots of ACEi/ARB association with the risk of COVID-19 infection and disease severity, in studies including only patients with **hypertension.**

Study or Subgroup	Weight	Odds Ratio IV, Random, 95% CI
**Positive test**		
Chodick 2020	15.6%	1.19 [0.96, 1.47]
Gnavi - DM/CVD 2020	6.8%	0.95 [0.68, 1.34]
Gnavi - Hypertension 2020	10.4%	0.78 [0.60, 1.02]
Mehta 2020	21.3%	0.97 [0.81, 1.15]
Rentsch 2020	13.9%	0.98 [0.78, 1.23]
Reynolds 2020	32.0%	1.02 [0.90, 1.16]
**Subtotal (95% CI)**	**100.0%**	**0.99 [0.91, 1.09]**
Heterogeneity: Tau² = 0.00; Chi² = 6.25, df = 5 (P = 0.28); I² = 20%		
Test for overall effect: Z = 0.12 (P = 0.90)		
**Mortality among COVID-19**		
Bean 2020	19.1%	1.23 [0.93, 1.62]
Chen 2020	3.0%	0.41 [0.12, 1.48]
Hu 2020	0.5%	3.93 [0.16, 98.06]
Huang 2020	0.6%	0.19 [0.01, 3.92]
Ip 2020	20.0%	0.66 [0.51, 0.85]
Jung 2020	12.2%	0.88 [0.54, 1.44]
Li 2020	10.5%	0.76 [0.44, 1.33]
Meng 2020	0.5%	0.47 [0.02, 12.14]
Montastruc 2020	2.4%	0.86 [0.20, 3.67]
Peng 2020	3.2%	1.32 [0.38, 4.52]
Tan 2020	0.7%	0.08 [0.00, 1.42]
Tedeschi 2020	16.2%	0.97 [0.68, 1.39]
Yang 2020	2.1%	0.32 [0.07, 1.51]
Zeng 2020	1.8%	0.65 [0.12, 3.58]
Zhang 2020	5.6%	0.37 [0.15, 0.89]
Zhou 2020	1.6%	0.49 [0.08, 2.97]
**Subtotal (95% CI)**	**100.0%**	**0.79 [0.62, 1.00]**
Heterogeneity: Tau² = 0.06; Chi² = 23.07, df = 15 (P = 0.08); I² = 35%		
Test for overall effect: Z = 1.97 (P = 0.05)		
**Severe/critical disease among COVID-19**		
Bean 2020	12.8%	0.63 [0.47, 0.84]
Feng 2020	3.5%	0.18 [0.06, 0.57]
Hu 2020	7.4%	1.17 [0.61, 2.26]
Huang 2020	1.0%	0.47 [0.05, 4.91]
Jung 2020	6.6%	1.03 [0.50, 2.13]
Li 2020	10.3%	1.11 [0.71, 1.73]
Mehta 2020	10.6%	1.64 [1.07, 2.51]
Meng 2020	2.7%	0.33 [0.08, 1.31]
Peng 2020	2.7%	0.94 [0.24, 3.61]
Rentsch 2020	8.5%	1.66 [0.94, 2.93]
Reynolds 2020	14.1%	0.99 [0.82, 1.21]
Tan 2020	3.1%	1.01 [0.29, 3.58]
Yang 2020	6.2%	0.73 [0.34, 1.58]
Zhang 2020	9.9%	0.65 [0.41, 1.04]
Zhou 2020	0.8%	0.14 [0.01, 2.21]
**Subtotal (95% CI)**	**100.0%**	**0.89 [0.70, 1.14]**
Heterogeneity: Tau² = 0.10; Chi² = 34.13, df = 14 (P = 0.002); I² = 59%		
Test for overall effect: Z = 0.92 (P = 0.36)		

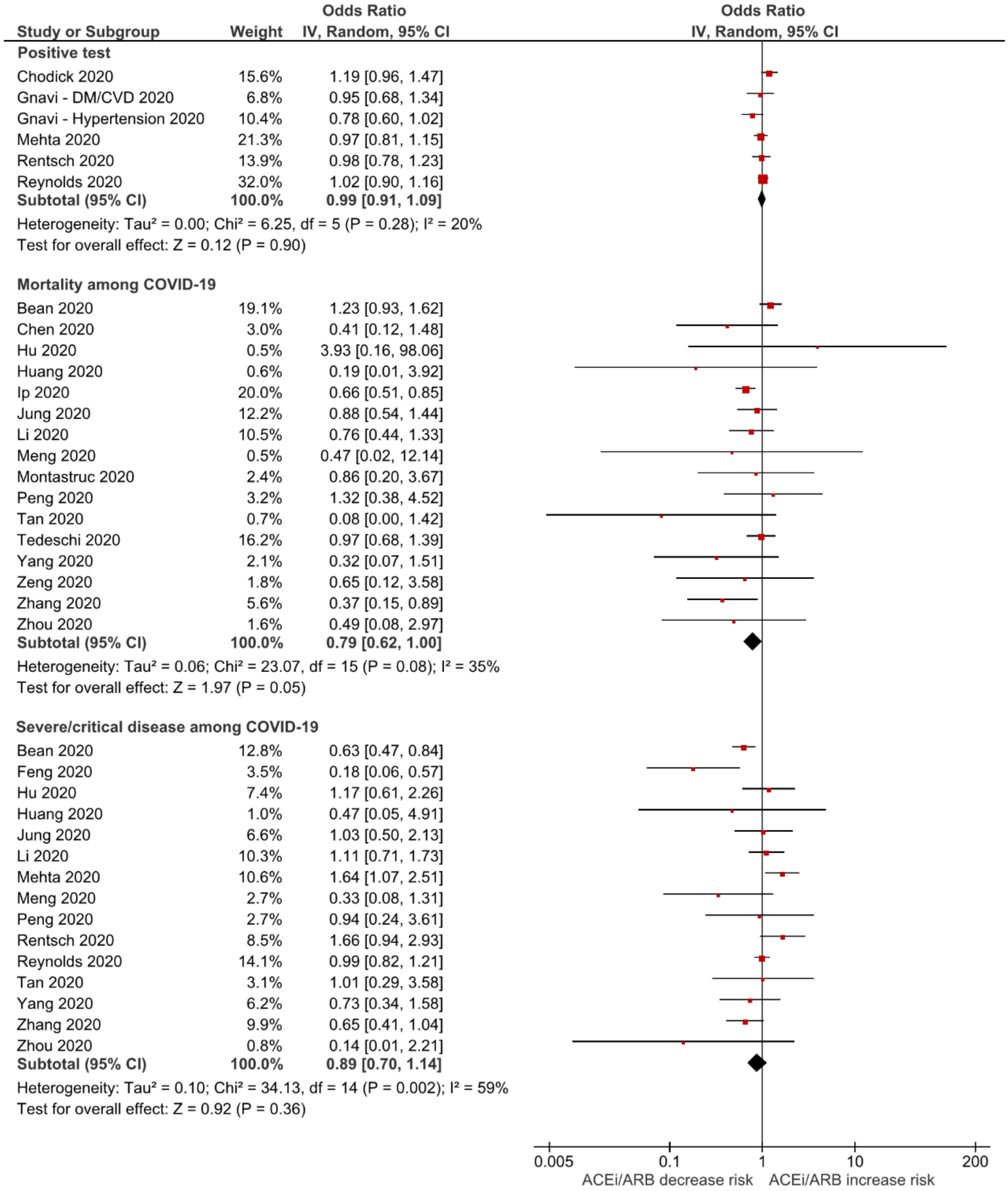

Supplementary Figure 7: Forest plots of ACEi/ARB association with the risk of COVID-19 infection and disease severity, **including unpublished data from medRxiv**.
